# Cyclin G2 Suppresses Glomerulosclerosis by Regulating Canonical Wnt Signalling

**DOI:** 10.1155/2018/6938482

**Published:** 2018-10-21

**Authors:** Chenyang Zhao, Jinlan Gao, Sen Li, Qi Liu, Xiaoyu Hou, Shenghuan Liu, Xuesha Xing, Manni Sun, Shusen Wang, Yang Luo

**Affiliations:** The Research Center for Medical Genomics, Key Laboratory of Cell Biology, Ministry of Public Health, Key Laboratory of Medical Cell Biology, Ministry of Education, College of Basic Medical Science, China Medical University, Shenyang, Liaoning, China

## Abstract

Recent data has shown that cyclin G2 (*CCNG2*) is an atypical cyclin that inhibits cell cycle progression and is often dysregulated in human cancers. The involvement of cyclin G2 in the occurrence and development of diabetic nephropathy (DN) has not been determined. In the present study, we conducted cyclin G2 knockout studies to determine whether this protein regulates glomerulosclerosis in DN mice. We found that cyclin G2 regulated the expression of renal glomerulosclerosis-related proteins via the canonical Wnt signalling pathway in glomerular mesangial cells. A cyclin G2 deficiency resulted in more severe renal injury in DN mice. These findings provided new insight into the pathogenesis of DN, revealing that cyclin G2 has a protective role in glomerulosclerosis and is a potential new target for the prevention and treatment of DN.

## 1. Introduction

Diabetic nephropathy (DN) is a relatively common chronic microvascular complication of diabetes that accounts for 30 to 40% of the morbidities of diabetic patients [1]. DN is also a leading cause of death in these patients [2]. The pathological features of DN include renal glomerular hypertrophy, thickening of the glomerular and tubular cell membranes and the basilar membrane, extracellular matrix accumulation, and, eventually, tubular interstitial fibrosis and glomerulosclerosis. The clinical manifestation of DN involves progressive, irreversible renal dysfunction and ultimately renal failure [3, 4].

Several cytokines and signalling pathways participate in the development of DN. The results from numerous studies have demonstrated that the TGF-*β*/Smad signalling pathway, a well-known profibrotic pathway in renal injury, plays a key role in the progression of DN. The PI3K/Akt, p38 MAPK, and JAK/STAT signalling pathways are also involved in the occurrence and development of DN [5, 6]. However, the pathogenesis of DN is not fully understood. Effective approaches for the treatment and prevention of DN are lacking. Thus, studies that address the molecular pathogenesis of DN are vital. Evidence suggests that the canonical Wnt signalling pathway participates in several processes involved in renal injury and glomerulosclerosis [3, 7]. Therefore, the canonical Wnt signalling may have a key role in the occurrence and development of DN.

The canonical Wnt signalling pathway is highly conserved and essential for foetal development, cell fate decisions, and tumour occurrence. Wnt signalling also controls physiological and pathological processes, such as angiogenesis, inflammatory reactions, and glomerulosclerosis [8]. The downstream proteins associated with this pathway are upregulated in the kidneys of patients with diabetes and diabetic mouse models [9, 10]. Similarly, high-glucose induction activates the canonical Wnt signalling pathway in glomerular podocytes and mesangial cells and causes excessive apoptosis of intrinsic renal cells [9, 11]. Suppression of this pathway improves proteinuria and renal fibrosis in patients with type 1 diabetes [12]. Recent findings have demonstrated that cyclin G2 has potent tumour-suppressive activity in epithelial ovarian cancer by inhibiting EMT through the attenuation of Wnt signalling [13]. The results from our preliminary studies suggested that cyclin G2 (*CCNG2*) could inhibit canonical Wnt signalling and regulate bone metabolism [14]. However, no evidence has shown whether cyclin G2 specifically functions in the occurrence and development of DN via this pathway.

The expression of cyclin G2 increases in cell cycle-arrested and terminally differentiated cells [15]. A large body of evidence indicates that cyclin G2 is an important contributor to suppress the development of cancer; the expression of cyclin G2 is downregulated in thyroid, oral, and breast cancer [16, 17]. In contrast, cyclin G2 overexpression in colonic cancer cells can induce p53-dependent G1/S-phase cell cycle arrest [15]. The biologic functions of cyclin G2 are mediated through its binding to protein phosphatase 2A (PP2A) and peroxisome proliferator-activated receptor *γ* (PPAR*γ*) [18, 19]. Recent findings suggest that cyclin G2 expression in the fatty tissues of obese patients is associated with steady-state carbohydrate metabolism [20]. However, in patients with type 2 diabetes, the levels of cyclin G2 are positively correlated with that of insulin-degrading enzyme (IDE) [20]. Moreover, insulin and insulin-like growth factor-1 (IGF-1) can significantly downregulate the expression of cyclin G2, thus stimulating DNA synthesis and promoting cell proliferation [21]. Taken together, these data suggest that cyclin G2 may be involved in the pathological processes of diabetes.

In this study, we investigated the regulatory role of cyclin G2 in the pathological processes of DN* in vitro* and* in vivo*. We found that cyclin G2 contributes to suppressing the occurrence and development of glomerulosclerosis in DN by regulating canonical Wnt signalling. Our results highlight a novel role for cyclin G2 as a potential target for amelioration of metabolic disease progression.

## 2. Materials and Methods

### 2.1. Cell Culture, Transfection, and Treatment

The human glomerular mesangial cell line (HMC) was purchased from ATCC (Manassas, VA) and cultured in DMEM containing 10% foetal bovine serum (Gibco, Life Technologies, Rochester, NY). Cells were transduced with lentivirus particles harbouring* CCNG2* or control GFP lentivirus (Genechem, Shanghai, China) and divided into low-glucose (LG; 5.5 mM D-glucose and 24.5 mM L-glucose) and high-glucose (HG; 30 mM D-glucose) treatment groups. For serum starvation, cells were grown in DMEM containing 1% serum for 24 h. For experiments involving the activation of canonical Wnt signalling, cells were treated with CHIR99021 (1 mM; B&D Systems, Minneapolis, MI) or DMSO control in DMEM for 72 h.

### 2.2. Western Blot Analysis

We evaluated the regulatory effects of cyclin G2 on the canonical Wnt signalling pathway and on the expression of proteins related to renal injury. Protein concentrations were detected with BCA (Life Technologies). Primary antibodies were anti–collagen IV, anti–cyclin D1, anti–*β*-tubulin, anti–phospho-*β*-catenin (Ser33/37/Thr41) (Cell Signalling Technology, Boston, MA), anti-GSK3*β*, anti–phospho-GSK3*β* (Ser9) (Santa Cruz Biotechnology), anti–*β*-catenin (Sigma-Aldrich), anti-fibronectin (FN), anti-MMP7, and anti–cyclin G2 (Proteintech, Chicago, IL). All experiments were performed in triplicate.

### 2.3. Mouse Model of Streptozotocin-Induced Diabetes

To investigate the* in vivo* functions of cyclin G2, we constructed* Ccng2*^−/−^ mice (Shanghai Model Organisms Center, China) and induced diabetes by injecting streptozotocin (STZ) into* Ccng2*^−/−^ and wild-type (WT; C57BL/6) mice. We assessed the effects of cyclin G2 knockout on the development of DN and blood glucose and urine protein content. In brief, male WT and* Ccng2*^−/−^ mice were housed in cages, fed with normal chow, and maintained on a 12-hour light-dark cycle. Diabetes was induced as described previously [22]. Eight-week-old mice received five consecutive intravenous injections of STZ (50 mg/kg; Sigma-Aldrich) in citrate buffer (pH 4.6) or citrate buffer only. A blood glucose level of >11.2 mmol/L was confirmed three days after STZ administration at three different time points. Mice were euthanised 16 weeks after STZ injection, and renal tissues were collected. All institutional and national guidelines for the care and use of laboratory animals were followed. All animal experiments were approved by the Animal Care and Use Committee of the Department of Animal Resources, China Medical University.

### 2.4. Morphological Studies

Renal tissue from* Ccng2*^−/−^ mice was examined 16 weeks after the induction of diabetes to determine the effects of cyclin G2 on renal injury and glomerulosclerosis. Specifically, renal tissue was fixed in 4% paraformaldehyde and embedded in paraffin. Sections (4 *μ*m) were examined using periodic acid-Schiff (PAS) and haematoxylin-eosin (HE) stains (Solarbio Life Sciences, Beijing, China). Fifteen randomly selected glomeruli were analysed per animal to evaluate glomerular size. The area of the glomerular tuft and the mesangial matrix index (MMI) were measured using Adobe Photoshop CS6. The MMI was defined as the PAS-positive area in the glomerular tuft region and was calculated from the following equation: MMI = (PAS-positive area)/(tuft area). The results are presented as the mean ± standard deviation (SD).

For immunohistochemistry, sections were deparaffinized, rehydrated, and autoclaved for 10 min in citrate buffer for antigen retrieval. Nonspecific binding was blocked by incubation for 30 min in 10% goat or rabbit serum. Samples were incubated with primary antibody (anti-*β*-catenin, anti-collagen IV, or anti-FN) at 4°C overnight. After washing in PBS, the sections were incubated with secondary antibody, and specific staining was visualised using the Ultrasensitive S-P Kit (streptavidin-peroxidase; Sigma-Aldrich).

### 2.5. Statistical Analysis

Data are presented as the mean ± SD. Statistical analysis was performed using Student's* t*-test for comparisons between two groups or with the paired* t-*test for comparison between more than two groups. Statistical significance was defined as* P *< 0.05.

## 3. Results

### 3.1. Cyclin G2 Inhibits the Expression of Tubular Glomerulosclerosis-Related Proteins in HMC Cells

**Figure 1:**
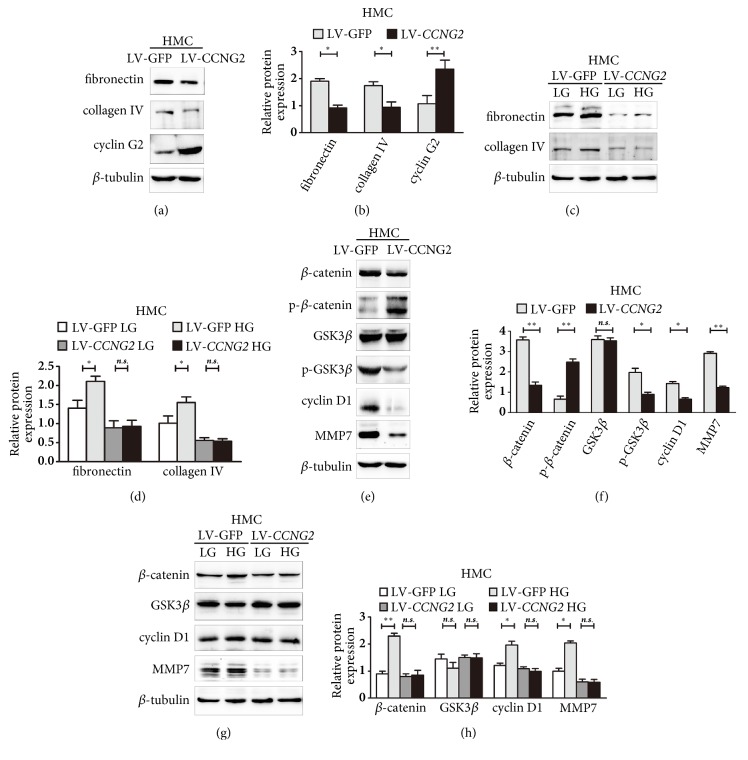
**Cyclin G2 inhibits the expression of glomerulosclerosis-related proteins and canonical Wnt signalling in HMC cells**. (a) Representative western blots and (b) densitometry for FN and collagen IV in HMC cells transduced with lentiviral particles harbouring* CCNG2 *(LV-*CCNG2*) or GFP (LV-GFP; control group). (c) Representative western blots and (d) densitometry for FN and collagen IV in* CCNG2-*overexpressing HMC cells exposed to 30 mM D-glucose (HG) or 5.5 mM D-glucose + 24.5 mM L-glucose (LG; control group) for 72 h. (e) Representative western blots and (f) densitometry for *β*-catenin, p-*β*-catenin, GSK3*β*, p-GSK3*β*, cyclin D1, and MMP7 in HMC transduced with lentiviral particles expressing* CCNG2 *(LV-*CCNG2*) or GFP (LV-GFP; control group). (g) Representative western blots and (h) densitometry for *β*-catenin, GSK3*β*, cyclin D1, and MMP7 in* CCNG2-*overexpressing HMC cells exposed to HG or LG for 72 h. Values are presented as the mean ± SD (n = 3); ^*∗*^*p*< 0.05, ^*∗∗*^*p*< 0.01,* n.s.*: not significant.

To evaluate the effect of cyclin G2 on glomerulosclerosis, we overexpressed cyclin G2 in HMC cells using lentiviral (LV-*CCNG2*) transduction and analysed the expression of glomerulosclerosis-related proteins. Compared to the control group (LV-GFP [i.e., green fluorescent protein]), the levels of FN and collagen IV were significantly downregulated in cyclin G2-overexpressing HMC cells (Figures 1(a) and 1(b)).

High-glucose levels in diabetes may accelerate renal injury in DN [23]. To evaluate whether cyclin G2 inhibited the expression of glomerulosclerosis-related proteins under high-glucose conditions, we cultured HMC cells in a high concentration of glucose for 72 h, which led to the upregulation of FN and collagen IV. Overexpression of cyclin G2 suppressed this high-glucose–induced upregulation (Figures 1(c) and 1(d)). These results demonstrated an antiglomerulosclerosis activity for cyclin G2 in the context of diabetes in HMC cells.

### 3.2. Cyclin G2 Suppresses the Canonical Wnt Signalling Pathway in HMC Cells

Ectopic expression of cyclin G2 in HMC cells inhibited the expression of *β*-catenin and its targets, cyclin D1 and MMP7. Cyclin G2 overexpression also upregulated the levels of phosphorylated (p)-*β*-catenin (Ser33/37/Thr41) and downregulated p-GSK3*β* (Ser9) levels (Figures 1(e) and 1(f)). When HMC cells were cultured in high glucose, the levels of *β*-catenin, cyclin D1, and MMP7 were induced. However, overexpression of cyclin G2 abolished this effect (Figures 1(g) and 1(h)). Therefore, cyclin G2 could suppress the activation of the canonical Wnt signalling pathway by high glucose in HMC cells.

### 3.3. Cyclin G2 Alters the Expression of Glomerulosclerosis-Related Proteins by Regulating Canonical Wnt Signalling

**Figure 2:**
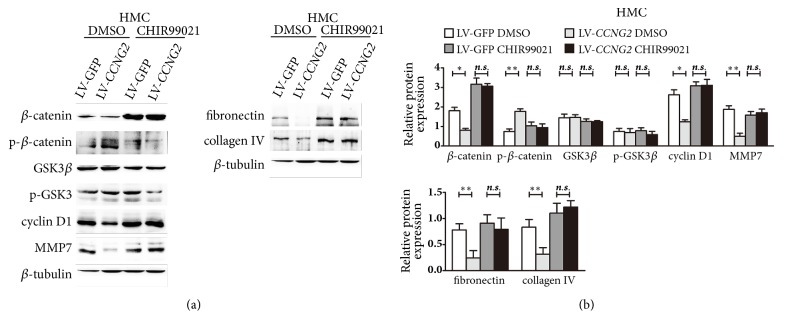
**Cyclin G2 inhibits the expression of proteins associated with glomerulosclerosis via canonical Wnt signalling**. (a) Representative western blots and (b) densitometry for *β*-catenin, p-*β*-catenin, GSK3*β*, p-GSK3*β*, cyclin D1, MMP7, FN, and collagen IV in cyclin G2-overexpressing HMC cells (LV-*CCNG2*) treated with 5 *μ*M CHIR99021 or DMSO (control group) for 72 h. Values are presented as the mean ± SD (n = 3); ^*∗*^*p < *0.05, ^*∗∗*^*p* < 0.01,* n.s.*: not significant.

To determine the regulatory mechanisms for cyclin G2 in renal glomerulosclerosis, we activated the canonical Wnt signalling pathway using the GSK3*β* inhibitor CHIR99021 [24]. In the absence of CHIR99021, cyclin G2 overexpression inhibited the expression of proteins associated with canonical Wnt signalling and glomerulosclerosis. CHIR99021 treatment abolished the effect of cyclin G2 overexpression on Wnt signalling and decreased its effect on FN and collagen IV levels (Figures 2(a) and 2(b)). These data suggested that cyclin G2 regulates the expression of glomerulosclerosis-related proteins via canonical Wnt signalling in HMC cells.

### 3.4. Cyclin G2 Deficiency Results in More Severe Renal Injury in DN Mice

**Figure 3:**
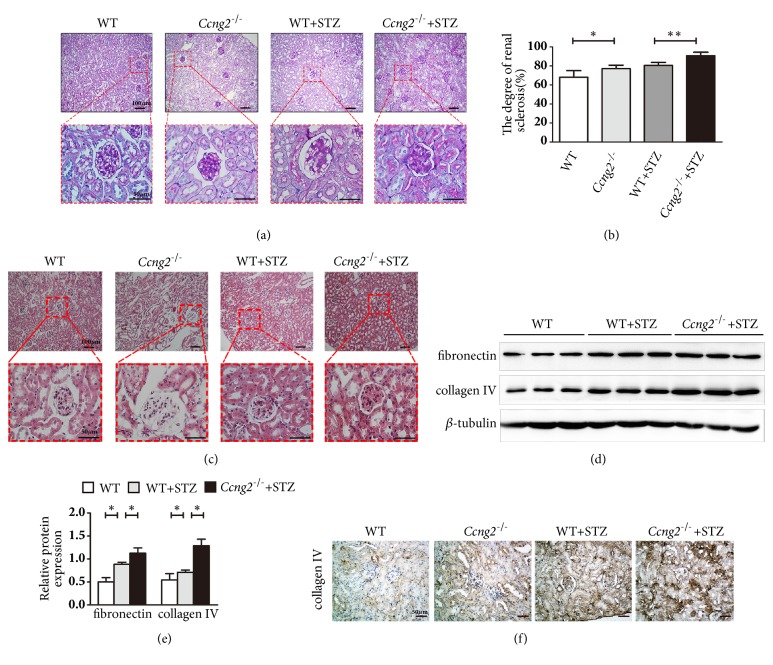
**Cyclin G2 deficiency increases renal injury in DN mice**. (a, b) PAS staining depicting the degree of renal sclerosis in control WT and* Ccng2*^−/−^ mice and STZ-induced WT and* Ccng2*^−/−^ DN mice (original magnification, 100×, 400×). (c) HE staining shows glomerular structures (original magnification, 100×, 400×). (d) Representative western blots and (e) densitometry for FN and collagen IV in the renal cortex of control WT and STZ-induced WT and* Ccng2*^−/−^ DN mice. (f) Representative collagen IV immunohistochemistry for control WT and* Ccng2*^−/−^ mice and STZ-induced WT and* Ccng2*^−/−^ DN mice (original magnification, 200×). Values are presented as the mean ± SD (n = 3); ^*∗*^*p< *0.05, ^*∗∗*^*p* < 0.01,* n.s.*: not significant.

Renal morphology and the severity of glomerulosclerosis in* Ccng2*^−/−^ and WT DN mice were evaluated by PAS and HE staining. Our results revealed that the glomerular basement membrane index was significantly increased in* Ccng2*^−/−^ DN mice compared to WT DN mice (Figures 3(a), 3(b), and 3(c)). In these DN renal tissues, the already elevated levels of FN and collagen IV were further increased following cyclin G2 knockout (Figures 3(d) and 3(e)). Increased collagen IV expression in the DN mouse kidney following cyclin G2 knockout was confirmed by immunohistochemistry (Figure 3(f)). Therefore, cyclin G2 appeared to ameliorate renal injury in a diabetes mouse model.

### 3.5. Cyclin G2 Deficiency Activates Canonical Wnt Signalling in the Kidneys of DN Mice

**Figure 4:**
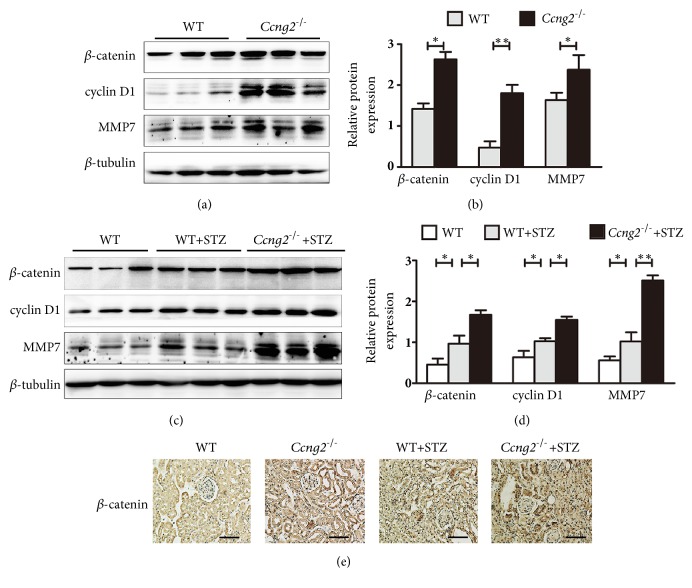
**A lack of cyclin G2 promotes the activation of canonical Wnt signalling in a DN mouse model**. (a) Representative western blots and (b) densitometry for *β*-catenin, cyclin D1, and MMP7 in the renal cortex of WT and* Ccng2*^−/−^ mice. (c) Representative western blots and (d) densitometry for *β*-catenin, cyclin D1, and MMP7 in the renal cortex of control WT and STZ-induced WT and* Ccng2*^−/−^ DN mice. (e) Representative *β*-catenin immunohistochemistry for control WT and* Ccng2*^−/−^ mice and STZ-induced WT and* Ccng2*^−/−^ DN mice (original magnification, 200×). Values are presented as the mean ± SD (n = 3); ^*∗*^*p< *0.05, ^*∗∗*^*p* < 0.01,* n.s.*: not significant.

We evaluated renal tissues of* Ccng2*^−/−^ and WT mice by immunohistochemistry and western blotting. We found that the levels of *β*-catenin, cyclin D1, and MMP7 in the renal cortex of* Ccng2*^−/−^ mice were higher than the levels in the renal cortex of WT mice (Figures 4(a) and 4(b)). These findings confirmed the regulatory effect of cyclin G2 on the components of the canonical Wnt signalling pathway* in vivo*.

To evaluate the effects of cyclin G2 inhibition on canonical Wnt signalling in diabetes, we assessed protein expression levels of Wnt signalling factors in the renal tissues of* Ccng2*^−/−^ and WT DN mice. We found that the levels of *β*-catenin, cyclin D1, and MMP7 in* Ccng2*^−/−^ DN mice were substantially higher than those of WT DN mice (Figures 4(c)–4(e)). Therefore, a lack of cyclin G2 resulted in enhanced canonical Wnt signalling in renal tissues of DN mice.

## 4. Discussion

DN has a complex pathogenesis that includes maladaptive metabolic mechanisms induced by high glucose and renal injury caused by an interaction of environmental and genetic factors [3, 25]. Involvement of cyclin G2 in the process of glomerulosclerosis in diabetes has not been reported previously. Other investigators have noted that cyclin G2 expression is negatively correlated with glucose and human insulin tolerance and regulates the metabolism of fatty tissues in internal organs [20]. Several authors have suggested that cyclin G2 interacts with PPAR*γ* to regulate lipogenesis [19]. In this study, we demonstrated that cyclin G2 knockout caused increased glomerular hypertrophy, accumulation of mesangial matrix, tuberous sclerosis, and other pathophysiological changes. Hence, cyclin G2 may be involved in the occurrence and development of DN.

Tubular mesangial cells control the glomerular filtration rate and provide capillary support. Notably, the accumulation of the mesangial matrix and formation of a mesangial extracellular matrix are essential features of DN [26]. By overexpressing cyclin G2 in HMC cells, we found that cyclin G2 significantly inhibited the expression of glomerulosclerosis-related proteins. Conversely, cyclin G2 knockout increased expression of glomerulosclerosis-related proteins in the mouse kidney.

Effective control of blood glucose may be sufficient to reduce the risks of diabetic complications. High-glucose treatment of HMC cells significantly upregulated the levels of glomerulosclerosis-related proteins. However, these high-glucose effects were suppressed by cyclin G2 overexpression. We observed increases in the content of accumulated mesangial matrix in* Ccng2*^−/−^ DN mice compared to WT DN mice. Glomerulosclerosis-related proteins were also elevated, and other pathophysiologic changes were evident. These results revealed a novel function of cyclin G2 as an inhibitor of renal injury progression in DN.

Canonical Wnt signalling is an evolutionarily conserved signal transduction pathway, which affects cellular events that modulate various disease processes and plays a critical role in kidney development [27]. High-glucose conditions and oxidative stress promote the activation of canonical Wnt signalling in the kidneys in diabetic animal models [12]. Investigators have demonstrated that the Huang Gan formula improved remnant renal function and alleviated glomerulosclerosis and tubulointerstitial fibrosis potentially by suppressing the canonical Wnt signalling pathway [28]. These results indicated that the canonical Wnt signalling pathway plays a vital role in DN. However, the precise regulatory mechanisms governing this process are unknown.

Recent studies suggested that cyclin G2 could inhibit canonical Wnt signalling in bone metabolism and epithelial ovarian cancer [13, 14]. In this study, overexpression of cyclin G2 in HMC cells resulted in the negative regulation of proteins involved in canonical Wnt signalling and suppressed the high-glucose–induced upregulation of these proteins. Conversely, cyclin G2 knockout increased the expression of canonical Wnt signalling factors in the mouse kidney and activated the canonical Wnt signalling pathway in the kidneys of DN mice. Therefore, cyclin G2 negatively regulates canonical Wnt signalling both* in vitro* and* in vivo*. Furthermore, treatment of HMC cells with the GSK3*β* inhibitor CHIR99021 (activator of Wnt signalling) abrogated the inhibitory effects of cyclin G2 on canonical Wnt signalling and glomerulosclerosis-related proteins.

## 5. Conclusions

A cyclin G2 deficiency precipitated the pathophysiological changes in mice with DN. Therefore, cyclin G2 is protective against DN progression, specifically against glomerulosclerosis. The regulation of the pathological progression in the DN mouse kidney by cyclin G2 occurred through its repression of canonical Wnt signalling. Our findings delineate a previously unidentified function of cyclin G2 as a protector in the pathologic progression of glomerulosclerosis in DN. These results provide potential new targets for the prevention and treatment of DN in the clinical setting.

## Data Availability

The data used to support the findings of this study are available from the corresponding author upon request.
