# Corrigendum to “Cyclin G2 Suppresses Glomerulosclerosis by Regulating Canonical Wnt Signalling”

**DOI:** 10.1155/2019/4104265

**Published:** 2019-04-01

**Authors:** Chenyang Zhao, Jinlan Gao, Sen Li, Qi Liu, Xiaoyu Hou, Shenghuan Liu, Xuesha Xing, Manni Sun, Shusen Wang, Yang Luo

**Affiliations:** The Research Center for Medical Genomics, Key Laboratory of Cell Biology, Ministry of Public Health, Key Laboratory of Medical Cell Biology, Ministry of Education, College of Basic Medical Science, China Medical University, Shenyang, Liaoning, China

In the article titled “Cyclin G2 Suppresses Glomerulosclerosis by Regulating Canonical Wnt Signalling” [1], there was an error in the Acknowledgments section where the grant numbers “(nos. LZDK201703 and 21601137)" should be corrected to (nos. LZDK201703 and 201601137)”. The Acknowledgments section should be updated as follows:

“This work was supported by the Foundation of the Education Department of Liaoning Province (nos. LZDK201703 and 201601137) and the National Natural Science Foundation of China (nos. 81400851, 81170543, and 81571440).”
